# Supplementary material for: A Dual Role of Heme Oxygenase-1 in Angiotensin II-Induced Abdominal Aortic Aneurysm in the Normolipidemic Mice
Source: Cells. 2021 Jan 15;10(1):163. doi: 10.3390/cells10010163 (PMC7830394; doi:10.3390/cells10010163)
Supplement: Supplementary file 1 [file cells-10-00163-s001.pdf]

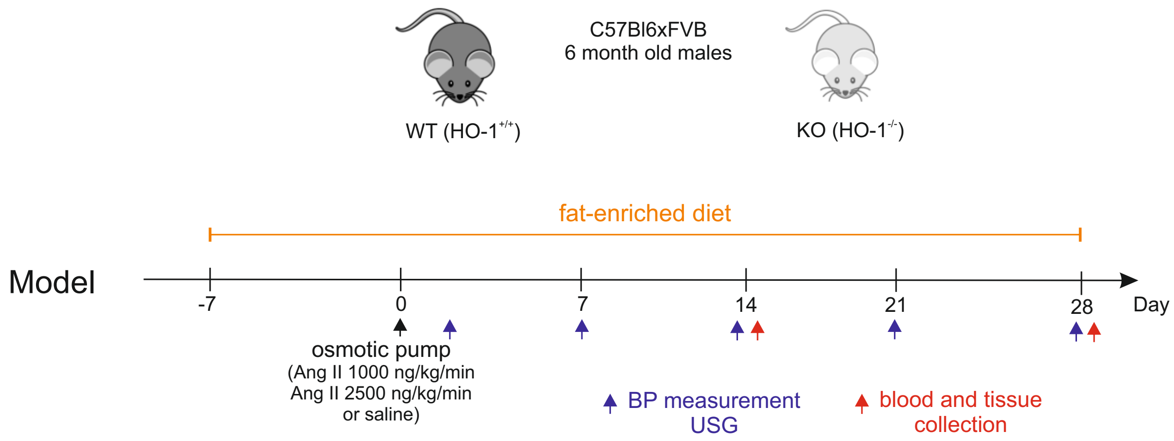
C57Bl6xFVB
6 month old males
WT (HO-1+/+)
KO (HO-1-/-)
fat-enriched diet
Model
-7
0
7
14
21
28
Day
osmotic pump
(Ang II 1000 ng/kg/min
Ang II 2500 ng/kg/min
or saline)
BP measurement
USG
blood and tissue
collection

Supp. Fig. 1

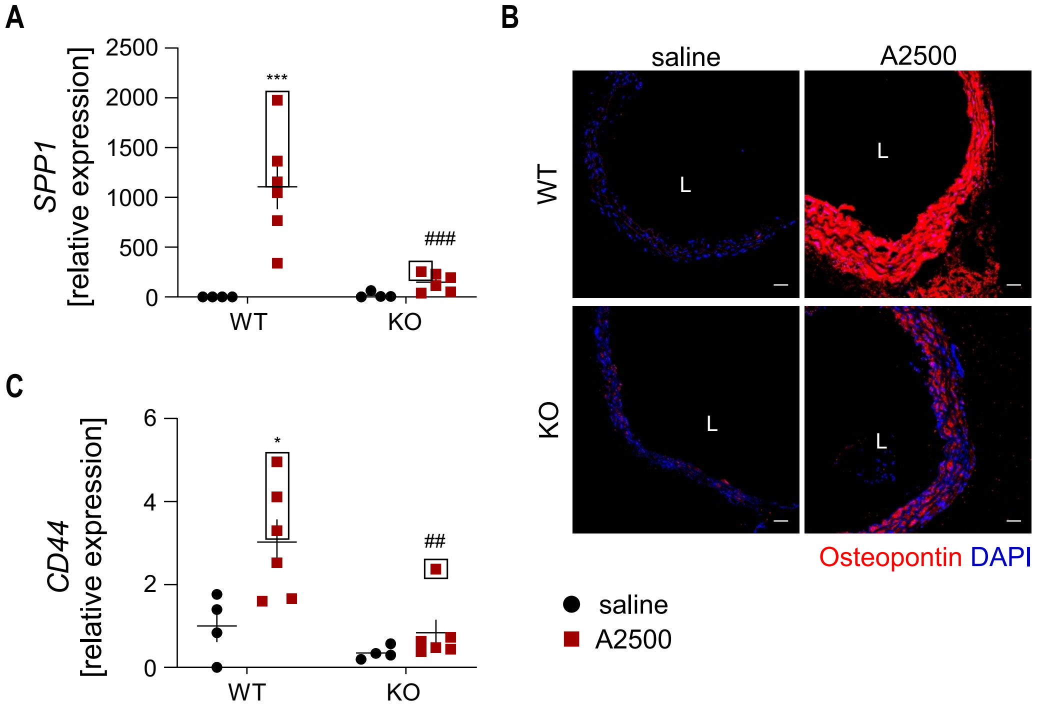

A
SPP1
[relative expression]
2500
2000
1500
1000
500
0
***
###
WT
KO
B
saline
A2500
WT
KO
L
Osteopontin DAPI
C
CD44
[relative expression]
6
4
2
0
*
##
WT
KO
saline
A2500

Supp. Fig. 2

Table. 1 The sequence of primers used in the study.

Gene	Primer sequence
Eef2	Forward: 5’- GACATCACCAAGGGTGTGCA -3’<br>Reverse: 5’- TCAGCACACTGGCATAGACC -3’
Hmox1	Forward: 5'- TTCTTCACCTTCCCCAACATTG -3'<br>Reverse: 5’- CAGCTCCTGCAACTCCTCAAA -3’
Vcam1	Forward: CCGGCATATACGAGTGTGAA<br>Reverse: GATGCGCAGTAGAGTGCAAG
Sele	Forward: ATGCCTCGCGCTTTCTCTC<br>Reverse: GTAGTCCCGCTGACAGTATGC
IL1β	Forward: 5’- CTGGTGTGTGACGTTCCCATTA-3’<br>Reverse: 5'- CCGACAGCACGAGGCTTT -3'
IL4	Forward: 5’- CTGGTGTGTGACGTTCCCATTA -3’<br>Reverse: 5'- CCGACAGCACGAGGCTTT -3'
IL6	Forward: 5’-AAAGAGTTGTGCAATGCAATGGCAATTCT-3’<br>Reverse: 5’- AAGTGCATCATCGTTGTTCATACA -3’
AT1R	Forward: 5'- ACCGCCCCTCAGATAACATG-3'<br>Reverse: 5’- AACCTGTCACTCCACCTCAGAAC -3’
AT2R	Forward: 5’- AACACTGGCAACTAAAAAGG -3’<br>Reverse: 5’- GAGATTATCAAAAGGACGGC -3’
ALAS1	Forward: 5’- TCTATATTTAGGGCCGCCAG-3’<br>Reverse: 5’- TCGGGATAAGAATGGGCATC -3’
FLVCR1	Forward: 5’- ATTGAATAAAATGCTCCAGTCATGA-3’<br>Reverse: 5’- ATCTGGAACCTGTGCAGAAACA-3’
SLC40A1	Forward: 5’-TCACCTAAAGATACTGAGCC -3’<br>Reverse: 5'- CTGGTTATAGTAGGAGACCC -3'
MKI67	Forward: 5'- GATTCCATTAACAAGAGTGAGG -3'<br>Reverse: 5'- CTCTTTTCAGTGGTGTATTAGG -3'
TGFβ1	Forward: 5’-TGATAAAGTGGAGTGAAGAGAG -3’<br>Reverse: 5’-GGCTTTTTGTAGTTTCCTAGAG -3’
TGFB2	Forward: 5’-CTCAAATAAGCCAGGGGGAAGG -3’<br>Reverse: 5'-TCCAGATTGCCACTGATTCCAA -3'
PAI	Forward: 5'- TGTCTTCAGCCCTTGCTTGCC -3'

	Reverse: 5'- CAGGCGTGTCAGCTCGTCTAC -3'
PLAU	Forward: 5'- AGAGTCTGAAAGTGACTATCTC-3'<br>Reverse: 5'- CCTTCGATGTTACAGATAAGC-3'
CD44	Forward: 5'- GAATTAGCTGGACACTCAAG -3'<br>Reverse: 5'- CACCTTCTCCTAACTATTGACC-3'
Col1α1	Forward: 5'- ACTACCGGGCCGATGATGCTAACG -3'<br>Reverse: 5'- CGATCCAGTACTCTCCGCTCTTCC -3'
Col1α2	Forward: 5'- GCCACCATTGATAGTCTCTCC -3'<br>Reverse: 5'- CACCCCAGCGAAGAACTCATA -3'
Col3α1	Forward: 5'- ATCTATGAATGGTGGTTTTCAGTT -3'<br>Reverse: 5'- TTTTGCAGTGGTATGTAATGTTCT -3'
MMP2	Forward: 5'- ACAGGACATTGTCTTTGATG -3'<br>Reverse: 5'- TACACAGCGTCAATCTTTTC – 3'
MMP3	Forward: 5'- CTAAAAGCATTCACACCCTG -3'<br>Reverse: 5'- TTTCTTCTCATCAAACCTCC -3'
MMP9	Forward: 5'- CTTCCAGTACCAAGACAAAG-3'<br>Reverse: 5'- ACCTTGTTCACCTATTTTG-3'
TIMP1	Forward: 5'-CATCCTCTTGTTGCTATCAC -3'<br>Reverse: 5'-CTGAATTTAGCCCTTATGACC -3'
TIMP2	Forward: 5'-GGATTCAGATGAGATCAAGC -3'<br>Reverse: 5'- GCCTTTCCTGCAATTAGATAC-3'
TIMP3	Forward: 5'-GCTAGAAGTCAACAAATACCAG -3'<br>Reverse: 5'-TAGTAGCAGGACTTGATCTTG -3'
